# LDL receptor-independent mechanisms of proprotein convertase subtilisin/kexin type 9 in cardiovascular pathophysiology

**DOI:** 10.3389/fcvm.2026.1744830

**Published:** 2026-03-16

**Authors:** Hang Su, Xunan Guo, Qiang Li, Xiaohang Yuan, Xiaolong Wu, Zihan Zhao, Yi Kan, Yifan Yang, Zhaolin Fu, Zhenrui Qi, Guangyuan Song

**Affiliations:** Interventional Center of Valvular Heart Disease, Beijing Anzhen Hospital, Capital Medical University, Beijing, China

**Keywords:** calcific aortic valve disease (CAVD), cardiomyocyte death, cardiovascular disease (CVD), efficacy gap, inflammation, low-density lipoprotein receptor (LDLR), proprotein convertase subtilisin/kexin type 9 (PCSK9), thrombosis

## Abstract

Proprotein convertase subtilisin/kexin type 9 (PCSK9) is a pivotal regulator of lipid metabolism and a validated therapeutic target in cardiovascular disease (CVD). While its canonical role in mediating low-density lipoprotein receptor (LDLR) degradation underpins its cholesterol-lowering effects, emerging evidence highlights diverse LDLR-independent actions that contribute to cardiovascular pathology. PCSK9 exerts pro-inflammatory, pro-atherosclerotic, pro-thrombotic, and cardiotoxic effects and promotes valvular calcification—thereby influencing vascular, myocardial, and structural heart disease beyond lipid regulation. This review delineates these non-canonical mechanisms, emphasizing PCSK9's roles in vascular inflammation, atherosclerosis, thrombosis, regulated cardiomyocyte death, and calcific aortic valve disease (CAVD). We also address key unresolved questions regarding the “efficacy gap” between pharmacological inhibition and lifelong genetic deficiency and examine the translational implications for next-generation inhibitors, including small molecules, vaccines, and gene-editing therapies. A deeper understanding of PCSK9's pleiotropic functions may inform precision strategies to achieve cardiovascular protection extending beyond LDL-C lowering.

## 1 Introduction

The identification of PCSK9 in 2003, initially described as neural apoptosis-regulated convertase 1 (NARC-1), marked a paradigm shift in the understanding of cholesterol homeostasis (1). Its critical role in cardiovascular biology was firmly established when gain-of-function (GOF) mutations in the PCSK9 gene were linked to autosomal dominant hypercholesterolemia, identifying it as the third causal locus after LDLR and APOB (2, 3). Conversely, natural loss-of-function (LOF) mutations were found to confer a profound cardioprotective phenotype, characterized by lifelong reductions in low-density lipoprotein cholesterol (LDL-C) and a dramatically lower (up to 88%) risk of coronary heart disease (4–6). These foundational genetic studies unequivocally validated PCSK9 as a premier therapeutic target.

The subsequent development of PCSK9 inhibitory therapies (PCSK9-iTs), including monoclonal antibodies (e.g., evolocumab, alirocumab) and small interfering RNA (inclisiran), has revolutionized the management of dyslipidemia and cardiovascular risk reduction. However, their collective relative risk reduction of approximately 15% for major adverse cardiovascular events (MACE) in FOURIER and ODYSSEY OUTCOMES, suggesting a potential “efficacy gap” compared to the profound (up to 88%) protection conferred by lifelong loss-of-function (LOF) mutations (7–10). This discrepancy has prompted a re-evaluation of PCSK9's biological functions. Accumulating evidence now indicates that beyond its canonical interaction with the LDLR, PCSK9 exerts multifaceted effects on the cardiovascular system in an LDLR-independent manner, influencing inflammatory, thrombotic, myocardial, immunologic, and valvular pathways.

This review synthesizes current evidence on the LDLR-independent roles of PCSK9 in distinct cell populations: endothelial cells (ECs), vascular smooth muscle cells (VSMCs), monocytes, macrophages, platelet, cardiomyocytes(CMs), and cardiac fibroblasts (CFs) and valvular interstitial cells(VICs) (Table 1). By clarifying the specific roles of these key cell types, we can better understand the mechanisms of vascular inflammation, atherosclerosis progression, myocardial remodeling and CAVD that persist even when systemic LDL-C is managed.By framing PCSK9 as a pleiotropic cardiovascular regulator rather than a lipid-centric molecule, we examine the mechanistic basis and clinical relevance of the efficacy gap between genetic deficiency and pharmacological inhibition and discuss implications for next-generation precision therapies aimed at extending cardiovascular protection beyond LDL-cholesterol lowering.

**Table 1:** LDLR-independent mechanisms of PCSK9 in different cell types.

Target cell type	Major PCSK9 receptors/interacting partners	Key signaling pathways	Principal cellular effects	Pathophysiological relevance
Endothelial cells (ECs)	LOX, ox-LDL, mtROS	NF-*κ*B, eNOS inhibition,	Endothelial dysfunction, reduced NO bioavailability, increased adhesion molecules (VCAM-1, ICAM-1)	Vascular inflammation, atherosclerosis initiation
	/	JNK/p38 MAPK	↑Bax/↓Bcl-2,caspase cleavage	Apoptosis promotion
	/	PI3K/AKT/mTOR	Suppressed cytoprotective autophagy	Autophagy suppression
	/	TGF-*β*/SMAD	EndMT(Snail/Slug expression; loss of VE-cadherin; gain of a-SMA/Vimentin)	Plaque fibrosis and calcification
Vascular smooth muscle cells (VSMCs)	/	MAPK/ERK, PI3K/AKT/mTOR	Phenotypic switching (contractile → synthetic), proliferation, migration,	Neointimal Hyperplasia, plaque progression,
	/	Osteogenic signaling(RUNX, BMP-2,ALPL), calcific extracellular vesicles(EVs)	Osteogenic differentiation	Vascular calcification
	ApoER2	Apoptosis signaling	VSMC senescence, apoptosis	Plaque vulnerability
Macrophages	TLR4,	MyD88/NF-κB	Pro-inflammatory activation, cytokine release (IL-1β, IL-6,TNF-α)	Amplification of vascular inflammation,atherosclerosis
	CAP1	Syk/PKCδ/NF-κB		
	CD36	/	Foam cell formation	
Platelets	CD36	p38 MAPK,cPLA2	Platelet activation, aggregation, thrombogenicity	Thrombosis, acute coronary events
Hepatocyte	LRP1	/	Elevated circulating FVIII	
Monocyte	TLR4	NF-κB	TF expression	Extracelluar coagulation initiation
Cardiomyocytes (CMs)	/	JNK/p38 MAPK	↑Bax/↓Bcl-2, caspase-3 activation, apoptosis	Excessive apoptosis
	mtROS	NLRP3 inflammasome	Caspase-1 activation, cleaves Gasdermin D (GSDMD), release mature IL-1β and IL-18	Pyroptosis
	TLR4	GPX4 suppression	Increasing the pool of labile iron and ROS, lipid Peroxidation	Ferroptosis
	LIAS,		Aggregation of lipoylated proteins and the proteotoxic stress	Cuproptosis
	KPNB1	ROS-ATM-LKB1-AMPK	Regulates mitophagy	Excessive autophagy
Cardiac fibroblasts (CFs)	TLR4	MyD88/NF-κB, NLRP3 inflammasome, TGF-β/SMAD, Notch1/Hes1, JAK2/STAT3	Myofibroblast activation, ECM deposition	Myocardial fibrosis, diastolic dysfunction
Valvular interstitial cells (VICs)	Lp(a), AVCAPIR,CD36	NF-κB, BMP2, RUNX2, Wnt/β-catenin	Osteogenic differentiation, calcific nodule formation	Calcific aortic valve disease (CAVD)

## 2 Biosynthesis and regulation of PCSK9

**Figure 1:**
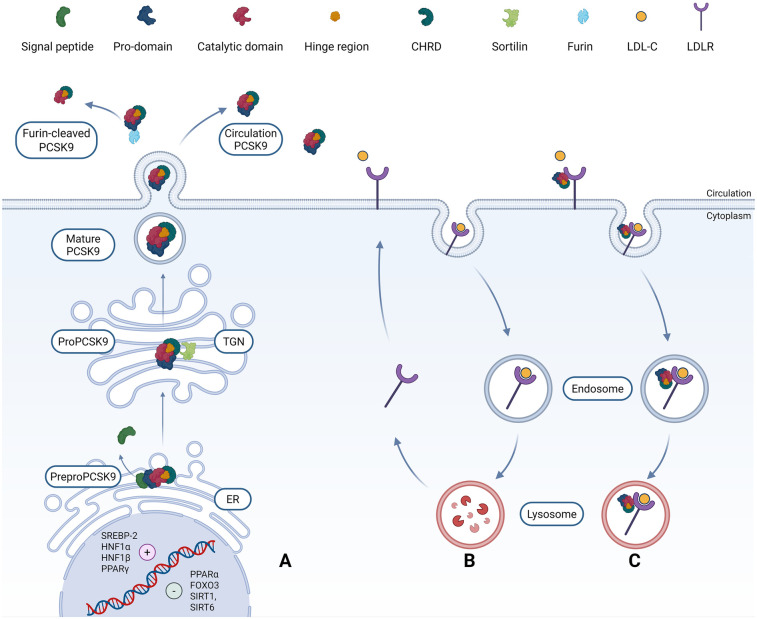
The biosynthesis, secretion, and molecular interaction mechanisms of PCSK9 with LDLR. **(A)** Synthesis and activation of PCSK9. The transcription of PCSK9 is regulated by nuclear factors (including SREBP-2, HNF1α/β, FoxO3, PPARα/γ, and SIRT1/SIRT6), and its translation occurs in the ER. The initial translation product is an inactive proenzyme form—preproPCSK9—which consists of five functional domains: the signal peptide, pre-domain, catalytic domain, hinge region, and CHRD. Within the ER, preproPCSK9 undergoes self-catalyzed cleavage to remove the signal peptide, resulting in proPCSK9. **(B)** Maturation and secretion pathway. ProPCSK9 is transported to the TGN, where it binds to sortilin and undergoes proteolytic processing to form a mature heterodimer. This dimer is then secreted into the circulatory system via the endosomal pathway. Mature PCSK9 typically binds to the prodomain in a non-covalent manner, in the circulation, it can be cleaved by furin, releasing a truncated peptide segment with reduced activity. **(C)** Mechanism of PCSK9-mediated LDLR degradation. Under physiological conditions, LDLR on the cell surface bind to LDL-C in the circulation, internalize via clathrin-coated pits, and degrade cholesterol particles through lysosomes, while LDLR itself can be recycled back to the cell membrane. The core pathological role of PCSK9 lies in disrupting this cycle: its catalytic domain specifically binds to the EGF-A of LDLR, forming a complex. This binding triggers the internalization of the LDLR-PCSK9 complex via clathrin-coated vesicles, followed by targeted transport to lysosomes for degradation, resulting in a significant reduction in the number of LDLR molecules on the cell surface and a sustained increase in serum LDL-C levels. Created with Biorender.com.

The 22 kb human PCSK9 gene is located on the short arm of chromosome 1p32.3. The gene has 12 exons and 11 introns. At the transcriptional level, the PCSK9 promoter contains an important region that is essential for transcription, called the sterol response element (SRE). Sterol response element binding protein (SREBP-1/2) is the main transcription factor that connects to the SRE promoter in PCSK9. Low dietary cholesterol concentrations upregulate the expression of SREBP-1/2, which in turn regulates the level of PCSK9 in the blood circulation (11). In addition, both hepatocyte nuclear factor (HNF1α/1β) can positively regulate the transcription of PCSK9.In contrast, forkhead box protein O3 (FOXO3) and deacetylases SIRT1, SIRT6 are negative regulators of PCSK9 transcription. Peroxisome proliferator-activated receptor gamma (PPARγ) increases gene expression of PCSK9, while PPARα decreases gene expression (12). The mRNA of human PCSK9 is 3,710 base pairs (bp) long over 12 exons and encodes a protein with 692 amino acids (aa).The protease is manufactured in the endoplasmic reticulum (ER) with a molecular mass of 120 kDa. It is secreted as an inactive protein and subsequently undergoes post-translational modifications to form a mature protein of 62 kDa.PreProPCSK9 consists of five segments: a signal peptide (aa 1–30), an N-terminal prodomain (aa 31–152), a catalytic domain containing the active site (aa 153–421), a hinge region (aa 422–452), and a cys/C-terminal cysteine-histidine-rich domain (CHRD) consisting of M1 (aa 453–529), M2 (aa 530–603), and M3 (aa 604–692) (13, 14) (Figure 1). Following its synthesis, PreProPCSK9 is directed to the endoplasmic reticulum by its signal peptide, which is subsequently cleaved by signal peptidase to generate ProPCSK9. An autocatalytic cleavage event then occurs within the catalytic domain, yielding mature PCSK9. In the mature form, the prodomain remains non-covalently bound to the catalytic domain, maintaining the protease in an inactive state. Finally, the mature PCSK9 is transported to the trans-Golgi network(TGN), where its interaction with Sortilin facilitates sorting, packaging into secretory vesicles, and eventual release into the circulation (14–16).

While hepatocytes are the primary source of circulating PCSK9, extrahepatic synthesis occurs in tissues including the intestine, kidneys, and nervous system (17). The canonical function of secreted PCSK9 entails its high-affinity binding to the epidermal growth factor-like repeat A (EGF-A) domain of the hepatic LDLR. This interaction redirects the LDLR-PCSK9 complex to lysosomal degradation, attenuating receptor recycling and consequently elevating plasma LDL-C levels—a process that is independent of PCSK9's enzymatic activity (18). The binding affinity of PCSK9 for the LDLR can be attenuated by furin-mediated cleavage of its prodomain (19). While the precise degradation mechanism remains incompletely defined, the PCSK9 CHRD and binding partners such as cyclase-associated actin cytoskeleton regulatory protein 1(CAP1) and major histocompatibility complex class I (MHC-I) are known to be crucial for directing the PCSK9-LDLR complex to lysosomal degradation. This degradative function extends to other LDLR family members possessing homologous EGF-like domains, including very low-density lipoprotein receptor(VLDLR), LDLR-related protein 1 (LRP1), and apolipoprotein E receptor2(ApoER2) (14, 20).

## 3 Pro-inflammatory and pro-atherosclerotic effect of PCSK9

**Figure 2:**
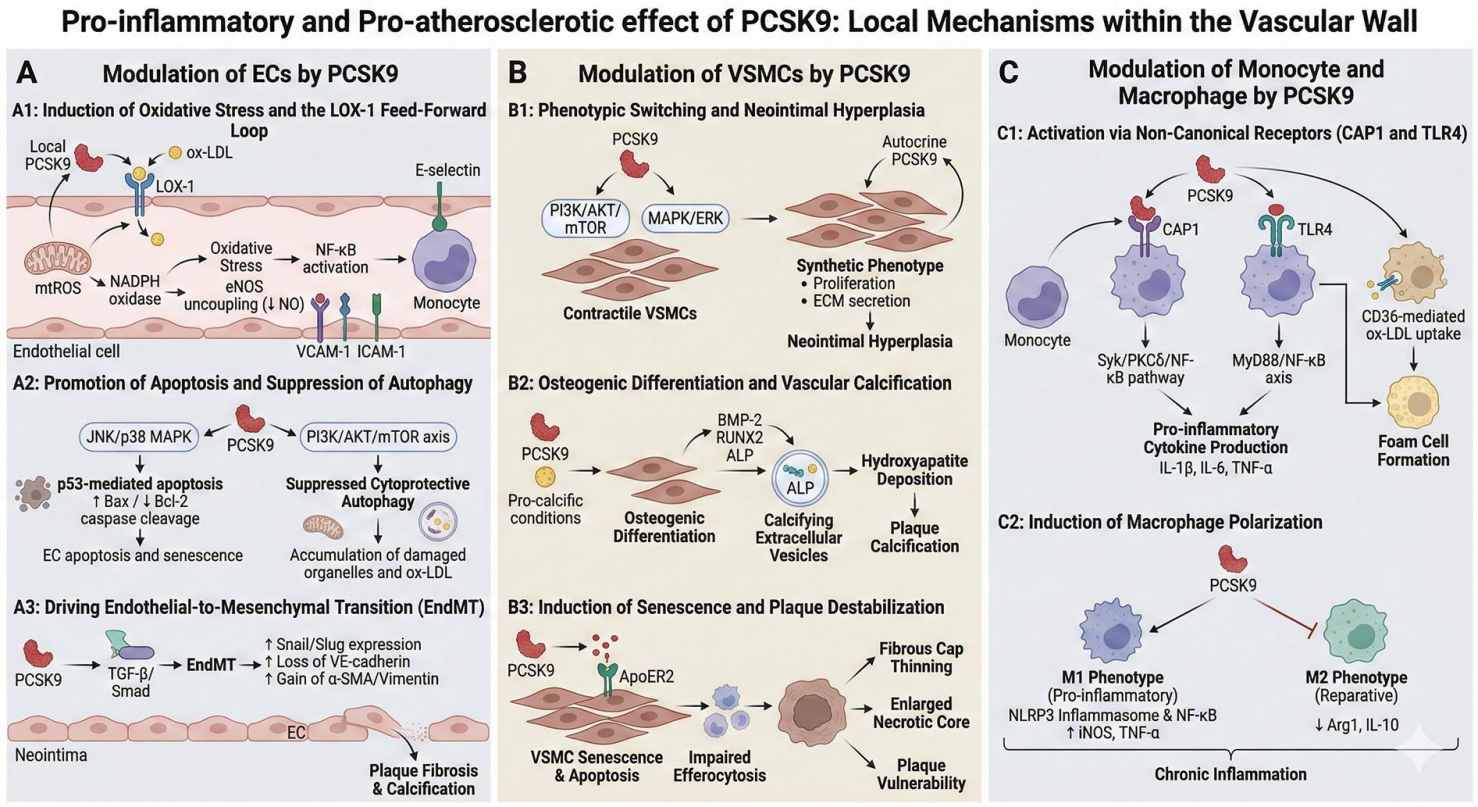
Pro-inflammatory and pro-atherosclerotic effect of PCSK9: local mechanisms within the vascular wall. **(A)** Modulation of Endothelial Cells (ECs) by PCSK9: (A1) Induction of Oxidative Stress and LOX-1 Loop: Local PCSK9 upregulation triggers a positive feedback loop with LOX-1. This facilitates ox-LDL uptake, activates NADPH oxidase, and increases mitochondrial ROS (mtROS), leading to NF-κB activation and the expression of adhesion molecules (VCAM-1, ICAM-1, E-selectin). (A2) Apoptosis and Autophagy Suppression: PCSK9 activates JNK/p38 MAPK pathways to induce p53-mediated apoptosis (increased Bax/decreased Bcl-2). Concurrently, it inhibits the PI3K/AKT/mTOR axis, suppressing cytoprotective autophagy and causing the accumulation of damaged organelles. (A3) Endothelial-to-Mesenchymal Transition (EndMT): PCSK9 promotes EndMT via the TGF-β/Smad signaling pathway, characterized by the upregulation of Snail/Slug, loss of VE-cadherin, and gain of mesenchymal markers (α-SMA/Vimentin), contributing to plaque fibrosis. **(B)** Modulation of Vascular Smooth Muscle Cells (VSMCs) by PCSK9: (B1) Phenotypic Switching: PCSK9 drives contractile VSMCs toward a synthetic phenotype via PI3K/AKT/mTOR and MAPK/ERK pathways, promoting proliferation, migration, and extracellular matrix (ECM) secretion. (B2) Osteogenic Differentiation: Under pro-calcific conditions, PCSK9 upregulates BMP-2, RUNX2, and ALP, promoting the release of calcifying extracellular vesicles and hydroxyapatite deposition. (B3) Senescence and Destabilization: PCSK9 degrades ApoER2, inducing VSMC senescence and apoptosis. This impairs efferocytosis and leads to fibrous cap thinning and necrotic core enlargement. **(C)** Modulation of Monocytes and Macrophages by PCSK9: (C1) Activation via Non-Canonical Receptors: PCSK9 binds to CAP1 and TLR4, triggering inflammatory signaling (Syk/PKCδ and MyD88/NF-κB pathways) and cytokine production (IL-1β, IL-6, TNF-α). It also enhances CD36-mediated ox-LDL uptake, fostering foam cell formation. (C2) Macrophage Polarization: PCSK9 acts as a molecular switch, promoting polarization toward the pro-inflammatory M1 phenotype while inhibiting the reparative M2 phenotype. Created with Biorender.com.

While the liver is the principal source of circulating PCSK9, it is now established that PCSK9 is also expressed locally within the vascular wall by endothelial cells (ECs), vascular smooth muscle cells (VSMCs), and macrophages (21). This local expression underpins a broad spectrum of LDLR -independent pleiotropic effects (13). Under physiological conditions, PCSK9 expression in quiescent vascular cells is minimal. However, in atherosclerosis, pro-inflammatory stimuli—such as lipopolysaccharide (LPS), tumor necrosis factor-α (TNF-α), oxidized LDL (ox-LDL), and disturbed shear stress—markedly upregulate PCSK9 expression in ECs, VSMCs, and macrophages (22, 23). The resulting local accumulation of PCSK9 within plaques can far exceed systemic levels, establishing a self-amplifying pathogenic feedback loop that sustains chronic inflammation (Figure 2). This provides a compelling rationale for considering PCSK9-ITs as a direct anti-inflammatory strategy at the tissue level.

### 3.1 Modulation of ECs by PCSK9

The endothelium serves as the primary interface between the circulating blood and the vascular wall. Far from being a passive barrier, endothelial cells are active participants in PCSK9-mediated pathology. PCSK9 functions as both a circulating ligand and an autocrine/paracrine factor produced by ECs under stress, driving dysfunction through three primary mechanisms: the amplification of oxidative stress via positive feedback loops, the induction of apoptosis, and the promotion of phenotypic transitions that fuel plaque progression.

#### 3.1.1 Induction of oxidative stress and the LOX-1 feed-forward loop

A central mechanism of PCSK9-induced endothelial dysfunction is its reciprocal regulation with Lectin-like Oxidized LDL Receptor-1 (LOX-1). Under homeostatic conditions, PCSK9 expression in ECs is minimal. However, pro-atherogenic stimuli, particularly ox-LDL, TNF-α, and disturbed shear stress, trigger a marked transcriptional upregulation of PCSK9 within the endothelium (24).

Elevated intracellular or local extracellular PCSK9 upregulates the transcription and surface expression of LOX-1, the primary receptor for ox-LDL on endothelial cells.1 This upregulation facilitates the massive influx of ox-LDL into the endothelial cytoplasm (25). Conversely, the binding of ox-LDL to LOX-1 stimulates further secretion of PCSK9, creating a self-perpetuating cycle.This mutual induction is mediated principally by mitochondrial reactive oxygen species (mtROS). Elevated PCSK9 enhances the activation of NADPH oxidase enzymes, specifically the NOX2 isoform, via redox-sensitive signaling pathways such as p38 mitogen-activated protein kinase (p38 MAPK). The resulting surge in ROS generation creates a “self-perpetuating oxidative stress loop” that amplifies vascular injury.

The oxidative environment inactivates endothelial nitric oxide synthase (eNOS), leading to reduced bioavailability of nitric oxide (NO) and impaired endothelium-dependent vasodilation, an early hallmark of atherosclerosis (26). Oxidative stress triggers the nuclear translocation of Nuclear Factor kappa B (NF-κB), which binds to the promoter regions of adhesion molecules. PCSK9 overexpression has been directly linked to increased surface expression of vascular cell adhesion molecule-1 (VCAM-1),intercellular adhesion molecule-1 (ICAM-1), and E-selectin (27). This facilitates the tethering, rolling, and diapedesis of monocytes into the sub-endothelial space.Silencing endothelial PCSK9 has been shown to restore the activity of SIRT1, an NAD+-dependent deacetylase associated with anti-aging and antioxidant protection, suggesting that PCSK9 actively represses this defense mechanism to maintain a pro-oxidant state.

#### 3.1.2 Promotion of apoptosis and suppression of autophagy

Beyond oxidative stress, PCSK9 directly compromises the structural integrity of the endothelium by promoting apoptosis. This effect is mediated through the activation of stress-activated protein kinases, including c-Jun N-terminal kinase (JNK) and p38 MAPK (28).

The analysis indicates that PCSK9 engages p53-mediated apoptotic pathways. Overactivity of endothelial PCSK9 leads to the upregulation of downstream targets such as p21^CIP1^ and p16^INK4a^. This induces cell cycle arrest at the G1/S checkpoint, driving ECs into a senescent state characterized by enlarged morphology and a “senescence-associated secretory phenotype” (SASP) analogous to that seen with CDK4/6 inhibition. In the presence of ox-LDL, PCSK9 shifts the balance of Bcl-2 family proteins: it downregulates the anti-apoptotic protein Bcl-2 while upregulating the pro-apoptotic effector Bax. This imbalance leads to mitochondrial outer membrane permeabilization, the release of cytochrome c, and the cleavage of caspase-9 and caspase-3, effectively executing the apoptotic program (24).

Concurrently, PCSK9 suppresses cytoprotective autophagy in ECs. Under physiological stress, autophagy serves to clear damaged organelles and protein aggregates. However, PCSK9 activates the PI3K/AKT/mTOR signaling axis, a potent inhibitor of autophagy. The inhibition of autophagic flux prevents the lysosomal clearance of ox-LDL and damaged mitochondria, exacerbating cellular toxicity and promoting endothelial erosion—a critical event that exposes the thrombogenic sub-endothelial matrix and precipitates acute coronary syndromes.

#### 3.1.3 Driving endothelial-to-mesenchymal transition (EndMT)

A critical, cell-type-specific mechanism by which endothelial cells contribute to atherosclerosis and fibrosis is EndMT. During EndMT, ECs lose their specific lineage markers (e.g., VE-cadherin, CD31/PECAM-1) and acquire mesenchymal characteristics (e.g., *α*-SMA, vimentin, fibroblast-specific protein 1), gaining migratory and invasive properties (29).

The evidence indicates that PCSK9 is a potent inducer of EndMT within atherosclerotic lesions. This process is driven by the upregulation of specific transcription factors, notably Snail, Slug, and Twist (29).

PCSK9 enhances the expression of Snail and Slug, likely through the sustained activation of the Transforming Growth Factor-beta (TGF-β) signaling pathway. TGF-β binding to its receptors phosphorylates Smad2/3, which form complexes with Smad4 and translocate to the nucleus to induce Snail expression. Snail then acts as a transcriptional repressor of VE-cadherin, leading to the disassembly of adherens junctions (30).

The induction of EndMT is amplified by low shear stress, a hemodynamic condition characteristic of atherosclerosis-prone regions (e.g., bifurcations). Low shear stress itself induces Snail expression, and this response is exacerbated by local PCSK9 accumulation, which is also upregulated by disturbed flow (31).

Additionally, the Notch and Wnt/β-catenin pathways are implicated in the PCSK9-mediated EndMT program. PCSK9 inhibition has been observed to suppress Wnt/β-catenin signaling, thereby attenuating the transition (30).

ECs undergoing EndMT delaminate from the luminal surface and migrate into the neointima. Once there, they differentiate into fibroblast-like cells or osteoblast-like cells, contributing significantly to plaque fibrosis and calcification (24). Furthermore, these transitioning cells become a local source of pro-inflammatory cytokines, fueling the inflammatory milieu of the plaque. Pharmacological blockade of PCSK9 has been shown to attenuate TGF-β-induced EndMT, preserving endothelial phenotype and reducing vascular fibrosis.

### 3.2 Modulation of VSMCs by PCSK9

VSMCs in the medial layer of the artery are not terminally differentiated; they retain significant phenotypic plasticity. PCSK9 acts as a critical modulator of this plasticity, driving VSMCs away from a contractile, quiescent state toward synthetic, osteogenic, or senescent phenotypes depending on the stage of vascular disease.

#### 3.2.1 Phenotypic switching and neointimal hyperplasia

In the early stages of vascular injury (e.g., following angioplasty) or atherosclerosis, VSMCs undergo a phenotypic switch to a “synthetic” state. This state is characterized by downregulation of contractile markers (e.g., SM22α, α-SMA) and upregulation of machinery for proliferation, migration, and extracellular matrix (ECM) secretion (32).

PCSK9 promotes this transition via the PI3K/AKT/mTOR signaling axes (33). High glucose and insulin resistance, which are known to upregulate PCSK9 in VSMCs, further amplify this pathway. Specifically, PCSK9 activation leads to the phosphorylation of ERK1/2, which drives the expression of proliferative genes and downregulates contractile markers (34).

The analysis suggests that mitochondrial dynamics play a crucial role in this switch. PCSK9-induced mtDNA damage and ROS production activate mTOR, which suppresses autophagy and promotes the synthetic phenotype (35). This metabolic reprogramming is essential to support the high energetic demands of proliferating cells.

Importantly, VSMCs are a significant source of PCSK9 within the vessel wall. Synthetic VSMCs secrete PCSK9, which acts in an autocrine manner to sustain proliferation and migration, contributing to neointimal hyperplasia. This autocrine loop suggests that VSMC-derived PCSK9 may be as relevant as circulating PCSK9 in the context of restenosis.

#### 3.2.2 Osteogenic differentiation and vascular calcification

Vascular calcification is a highly regulated process resembling bone formation (osteogenesis), involving the transdifferentiation of VSMCs. PCSK9 is a potent driver of VSMC calcification, a phenomenon particularly relevant in patients with chronic kidney disease (CKD) and advanced atherosclerosis.

Under pro-calcific conditions (e.g., high phosphate environments typical of CKD), PCSK9 expression is elevated in VSMCs. This intracellular PCSK9 promotes the transdifferentiation of VSMCs into osteoblast-like cells. Mechanistically, PCSK9 upregulates pro-calcific markers such as bone morphogenetic protein-2 (BMP-2), the master osteogenic transcription factor RUNX2, and alkaline phosphatase (ALP). Simultaneously, it downregulates anti-calcific proteins like matrix gla protein (MGP) and osteopontin (36).

A key insight from the analysis is the role of extracellular vesicles in this process. PCSK9-overexpressing VSMCs release specific “calcifying extracellular vesicles(EVs)” loaded with calcium, ALP, and tetraspanins (CD63, CD9) (36). These vesicles are secreted into the extracellular matrix, where they act as nucleation sites for the deposition of hydroxyapatite crystals. This suggests that PCSK9 drives calcification not just by altering gene expression, but by modifying the VSMC secretome to favor mineralization.

#### 3.2.3 Induction of senescence and plaque destabilization

In advanced atherosclerotic plaques, the role of PCSK9 in VSMCs shifts from promoting proliferation to inducing senescence and apoptosis. This duality is critical for understanding plaque vulnerability and rupture.

ApoER2 Degradation Mechanism: PCSK9 binds to and degrades Apolipoprotein E Receptor 2 (ApoER2) on the surface of VSMCs (37). ApoER2 is essential for transmitting survival signals and maintaining the contractile phenotype. Its PCSK9-mediated loss leads to VSMC polyploidization, a hallmark of senescence, and eventual apoptosis.

Consequences for the Fibrous Cap: VSMCs are the primary producers of collagen in the fibrous cap of the plaque. The PCSK9-induced loss of viable VSMCs via senescence and apoptosis reduces collagen synthesis and structural support, leading to cap thinning. Combined with the accumulation of apoptotic bodies (due to defective efferocytosis, which is also hindered by PCSK9), this creates a necrotic core prone to rupture (38, 39). Thus, PCSK9 acts as a destabilizing factor in advanced atherosclerosis, transitioning stable plaques into vulnerable lesions.

### 3.3 Modulation of monocyte and macrophage by PCSK9

Macrophages are the central architects of atherosclerotic inflammation and myocardial immune responses. The analysis of the provided data underscores that PCSK9 regulates macrophage biology through multiple non-canonical receptors and pathways, significantly influencing polarization, foam cell formation, and the specific ontogeny of cardiac macrophages.

#### 3.3.1 Activation via non-canonical receptors (CAP1 and TLR4)

While the LDLR mediates cholesterol uptake, PCSK9's pro-inflammatory effects in macrophages are largely LDLR-independent, mediated instead by CAP1 and TLR4.

Recently, PCSK9 CHRD has been reported to have structural homology with the human resistin, which is a pro-inflammatory cytokine inducing atherosclerosis. Identified as a high-affinity receptor for the CHRD of PCSK9, CAP1 is critical for PCSK9-induced inflammation (40, 41). The binding of PCSK9 to CAP1 on the surface of monocytes/macrophages triggers a signaling cascade involving the phosphorylation of spleen tyrosine kinase(Syk) and protein kinase C delta(PKCδ) (42). This pathway activates NF-κB, leading to the transcription of pro-inflammatory cytokines [interleukin-1β(IL-1β), IL-6, TNF-α] independent of LDL uptake.

PCSK9 acts as a damage-associated molecular pattern (DAMP) or sensitizer for TLR4 (43). It upregulates TLR4 expression and promotes its dimerization, enhancing the cell's sensitivity to other ligands like LPS or ox-LDL (44). This interaction activates the MyD88-dependent pathway, culminating in NF-*κ*B nuclear translocation. Importantly, inhibitors of the TLR4/NF-*κ*B axis (e.g., TAK-242) abolish PCSK9-induced cytokine production, confirming the specificity of this pathway (45).

PCSK9 also interacts with the scavenger receptor CD36, enhancing the uptake of ox-LDL (42). This promotes lipid accumulation and the transformation of macrophages into foam cells, the hallmark of the fatty streak.

#### 3.3.2 Induction of macrophage polarization

PCSK9 acts as a molecular switch in macrophage polarization, inducing the population toward a pro-inflammatory M1 phenotype and inhibiting the reparative M2 phenotype (46).

Through the activation of TLR4/NF-κB and the NLRP3 inflammasome, PCSK9 drives the expression of M1 markers such as inducible Nitric Oxide Synthase (iNOS), TNF-α, and Monocyte Chemoattractant Protein-1 (MCP-1) (47, 48). This creates a sustained inflammatory environment within the plaque and the myocardium.

Conversely, PCSK9 suppression (via genetic knockout or pharmacological inhibition) facilitates polarization toward the M2 phenotype (characterized by Arg1, CD206 expression), which is associated with tissue repair, fibrosis resolution, and anti-inflammatory cytokine release (e.g., IL-10) (46). This repolarization is a key mechanism underlying the potential benefits of PCSK9 inhibition in the recovery phase of acute myocardial infarction (AMI).

## 4 From plaque to clot: the prothrombotic role of PCSK9

**Figure 3:**
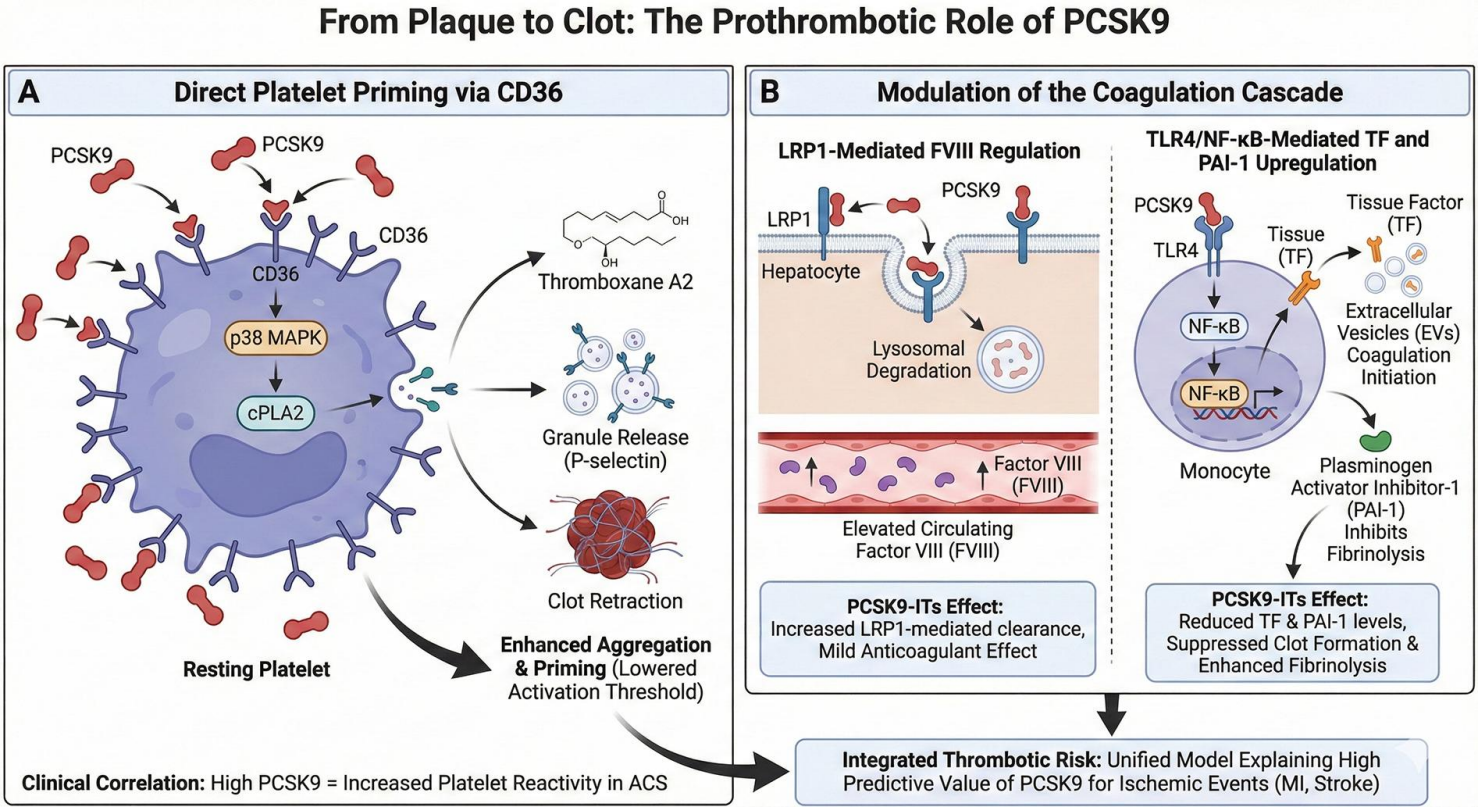
From plaque to clot: the prothrombotic role of PCSK9. **(A)** Direct Platelet Priming via CD36: PCSK9 binds directly to the CD36 receptor on resting platelets. This interaction activates p38 MAPK and cytosolic phospholipase A2 (cPLA2), leading to increased Thromboxane A2 generation, granule release (e.g., P-selectin), and enhanced clot retraction. This “primes” platelets, lowering the threshold for aggregation in response to agonists. B. Modulation of the Coagulation Cascade: LRP1-Mediated FVIII Regulation: PCSK9 promotes the lysosomal degradation of LRP1 in hepatocytes, reducing the clearance of Factor VIII (FVIII) and elevating its circulating levels. TLR4/NF-κB-Mediated TF and PAI-1 Upregulation: In monocytes, PCSK9 signaling via TLR4/NF-κB upregulates Tissue Factor (TF) and Plasminogen Activator Inhibitor-1 (PAI-1), thereby initiating coagulation and inhibiting fibrinolysis. PCSK9 inhibition (PCSK9-ITs) reverses these effects, offering potential antithrombotic benefits. Created with Biorender.com.

The clinical sequelae of atherosclerosis—myocardial infarction and ischemic stroke—are often precipitated by acute thrombosis. Accumulating evidence positions PCSK9 as a direct molecular link between chronic vascular disease and acute thrombotic events, influencing both cellular and soluble components of the coagulation system (Figure 3).

### 4.1 Direct platelet priming via CD36

A pivotal mechanism involves the direct binding of PCSK9 to the scavenger receptor CD36 on platelets, an interaction independent of LDL particles or LDLR (49). This engagement does not typically initiate aggregation but potently “primes” platelets, lowering their activation threshold for agonists like ADP and collagen. The PCSK9-CD36 interaction triggers an intracellular signaling cascade involving p38 MAPK and cytosolic phospholipase A2 (cPLA2), amplifying thromboxane A2 generation and enhancing aggregation, granule release (e.g., P-selectin), and clot retraction (50, 51). Clinically, this is reflected in the correlation between high PCSK9 levels and increased platelet reactivity in patients with acute coronary syndromes (52).

### 4.2 Modulation of the coagulation cascade

Beyond platelets, PCSK9 modulates soluble coagulation and fibrinolytic systems. It may elevate circulating Factor VIII (FVIII) levels by promoting the degradation of its clearance receptor, LRP1. Thus, PCSK9-ITs could potentially lower FVIII via increased LRP1-mediated clearance, exerting a mild anticoagulant effect (53). Furthermore, PCSK9 upregulates tissue factor (TF) expression on monocytes and EVs through TLR4/NF-*κ*B signaling, directly linking its pro-inflammatory action to coagulation initiation (45). PCSK9-ITs has been shown to reduce both TF and plasminogen activator inhibitor-1 (PAI-1) levels, suggesting a dual benefit of suppressing clot formation and enhancing fibrinolysis (54).

### 4.3 Integrated thrombotic risk: a unified model

PCSK9 establishes a unified model of atherothrombotic risk by concurrently accelerating plaque progression through inflammatory mechanisms and enhancing thrombotic susceptibility via platelet priming and coagulation factor regulation. This dual pathway explains the high predictive value of PCSK9 for ischemic events and underscores the efficacy of PCSK9-ITs in high-risk patients, as demonstrated in outcomes trials like ODYSSEY OUTCOMES, particularly following acute coronary syndrome.

## 5 Modulation of CMs by PCSK9

**Figure 4:**
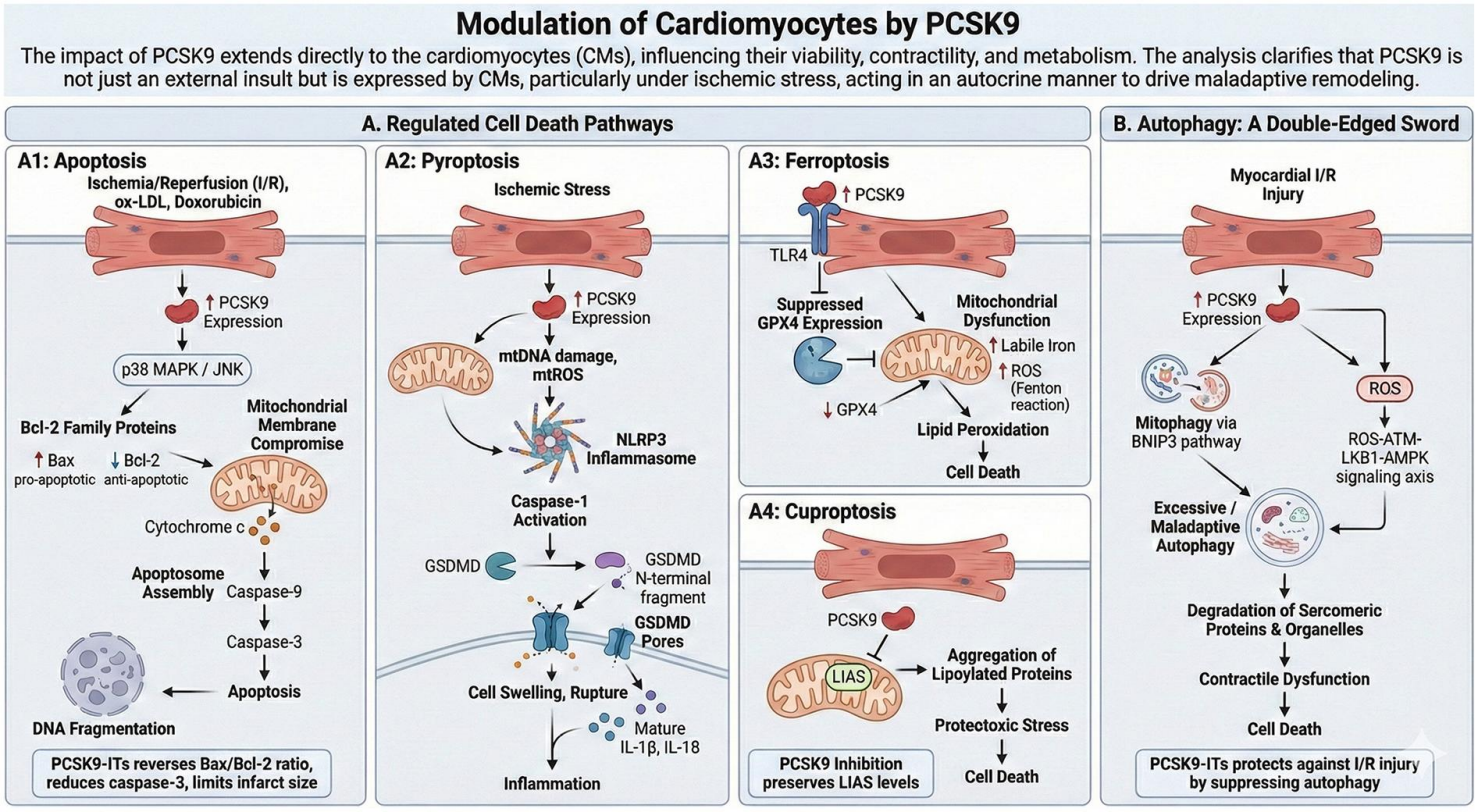
Modulation of cardiomyocytes (CMs) by PCSK9. **(A)** Regulated Cell Death Pathways: (A1) Apoptosis: Ischemia/Reperfusion (I/R) or ox-LDL stress upregulates PCSK9, which activates p38 MAPK/JNK. This shifts the balance of Bcl-2 family proteins (high Bax/low Bcl-2), compromising mitochondrial membrane integrity and activating the Caspase-9/Caspase-3 apoptotic cascade. (A2) Pyroptosis: PCSK9 acts as a primer for the NLRP3 inflammasome by promoting mtDNA damage and mtROS generation. This leads to Caspase-1 activation and Gasdermin D (GSDMD) pore formation, resulting in cell rupture and inflammatory cytokine release (IL-1β, IL-18). (A3) Ferroptosis: PCSK9 exacerbates ferroptosis by inhibiting GPX4 expression via TLR4 and inducing mitochondrial dysfunction, which increases labile iron and ROS (Fenton reaction), leading to lethal lipid peroxidation. (A4) Cuproptosis: PCSK9 targets Lipoyl Synthase (LIAS), causing the aggregation of lipoylated proteins and proteotoxic stress. **(B)** Autophagy: A Double-Edged Sword: PCSK9 dysregulates autophagic flux during myocardial I/R injury. It promotes maladaptive autophagy and mitophagy via the ROS-ATM-LKB1-AMPK axis and BNIP3 pathway, leading to the excessive degradation of sarcomeric proteins and organelles, contributing to contractile dysfunction and cell death. Created with Biorender.com.

The impact of PCSK9 extends directly to the CMs, influencing their viability, contractility, and metabolism. The analysis clarifies that PCSK9 is not just an external insult but is expressed by CMs, particularly under ischemic stress, acting in an autocrine manner to drive maladaptive remodeling (Figure 4).

### 5.1 Regulated cell death pathways

PCSK9 acts as a “master regulator” of multiple forms of programmed cell death in cardiomyocytes, contributing to the loss of functional myocardium in ischemic heart disease and heart failure.

#### 5.1.1 Apoptosis

PCSK9 acts as a critical switch in the intrinsic (mitochondrial) apoptotic pathway, particularly under conditions of oxidative or ischemic stress (55).

In cardiomyocytes exposed to stimuli such as ischemia/reperfusion(I/R), ox-LDL, or doxorubicin, PCSK9 expression is markedly upregulated. This upregulation shifts the balance of Bcl-2 family proteins by increasing the expression of the pro-apoptotic protein Bax and suppressing the anti-apoptotic protein Bcl-2. This increased Bax/Bcl-2 ratio is a definitive commitment step toward cell death.

The imbalance in Bcl-2 family proteins compromises mitochondrial membrane integrity, leading to the release of cytochrome c into the cytosol. This event triggers the assembly of the apoptosome and the activation of Caspase-9, which subsequently cleaves and activates the executioner Caspase-3. The activation of this caspase cascade leads to DNA fragmentation and the morphological hallmarks of apoptosis (56, 57).

This apoptotic program is regulated upstream by the activation of stress-activated protein kinases. PCSK9 promotes the phosphorylation of p38 MAPK and JNK. These kinases stabilize p53 and directly phosphorylate Bcl-2 family members to promote apoptosis.

In doxorubicin-induced cardiotoxicity, PCSK9 has additionally been reported to interact with importin subunit beta-1 (KPNB1), suggesting involvement in stress-induced nuclear signaling.

In models of doxorubicin-induced cardiotoxicity and myocardial infarction, PCSK9-ITs has been shown to reverse the Bax/Bcl-2 ratio, reduce the expression of caspase-3, and significantly limit infarct size and myocardial fibrosis, confirming the pivotal role of PCSK9 in driving cardiomyocyte apoptosis (58, 59).

#### 5.1.2 Pyroptosis

Pyroptosis is a highly inflammatory form of cell death driven by the inflammasome.

PCSK9 acts as a primer and activator for the NLRP3 inflammasome in CMs (55). Ischemic stress induces PCSK9 expression, which in turn promotes mitochondrial DNA (mtDNA) damage and the release of mtROS (60). These signals are sensed by NLRP3, leading to its assembly and the activation of Caspase-1.

Active Caspase-1 cleaves Gasdermin D (GSDMD) into its N-terminal fragment, which oligomerizes to form pores in the sarcolemma (60). This leads to cell swelling, rupture, and the release of mature IL-1β and IL-18, propagating inflammation to neighboring fibroblasts and immune cells.

#### 5.1.3 Ferroptosis

Ferroptosis is an iron-dependent form of cell death characterized by lipid peroxidation.

PCSK9 exacerbates ferroptosis by dysregulating the Glutathione Peroxidase 4 (GPX4) axis (55). Via TLR4 signaling, PCSK9 suppresses GPX4 expression, the primary enzyme responsible for neutralizing lipid peroxides (61, 62).

Concurrently, PCSK9 induces mitochondrial dysfunction, increasing the pool of labile iron and ROS (via the Fenton reaction). The combination of reduced antioxidant defense (GPX4) and increased oxidant burden leads to catastrophic lipid peroxidation of the sarcolemma and cell death (55).

#### 5.1.4 Cuproptosis

Recent evidence identifies PCSK9 as a regulator of cuproptosis, a copper-dependent cell death pathway (63).

PCSK9 targets Lipoyl Synthase (LIAS), a key enzyme involved in the lipoylation of mitochondrial TCA cycle proteins (64). PCSK9 inhibition preserves LIAS levels, preventing the aggregation of lipoylated proteins and the proteotoxic stress that characterizes cuproptosis. This interaction highlights a novel metabolic vulnerability induced by PCSK9 in the ischemic heart.

### 5.2 Autophagy: a double-edged sword

PCSK9 dysregulates autophagic flux in cardiomyocytes. While basal autophagy is protective, the form of autophagy induced by PCSK9 during I/R is maladaptive (65, 66).

PCSK9 is upregulated in the myocardial I/R injury hearts and regulates mitophagy via the Bcl-2/adenovirus E1B 19-kDa interacting protein (BNIP3) pathway, which in turn contributes to reperfusion injury after myocardial infarction. PCSK9-ITs protects against myocardial I/R injury by suppressing autophagy (66).

PCSK9 also promotes autophagy via the ROS-ATM-LKB1-AMPK signaling axis (65). By preventing the recycling of LDLR, PCSK9 may alter membrane lipid composition, affecting autophagosome formation.Excessive autophagy in this context leads to the degradation of essential sarcomeric proteins and organelles, contributing to contractile dysfunction and cell death.

## 6 Modulation of CFs by PCSK9

**Figure 5:**
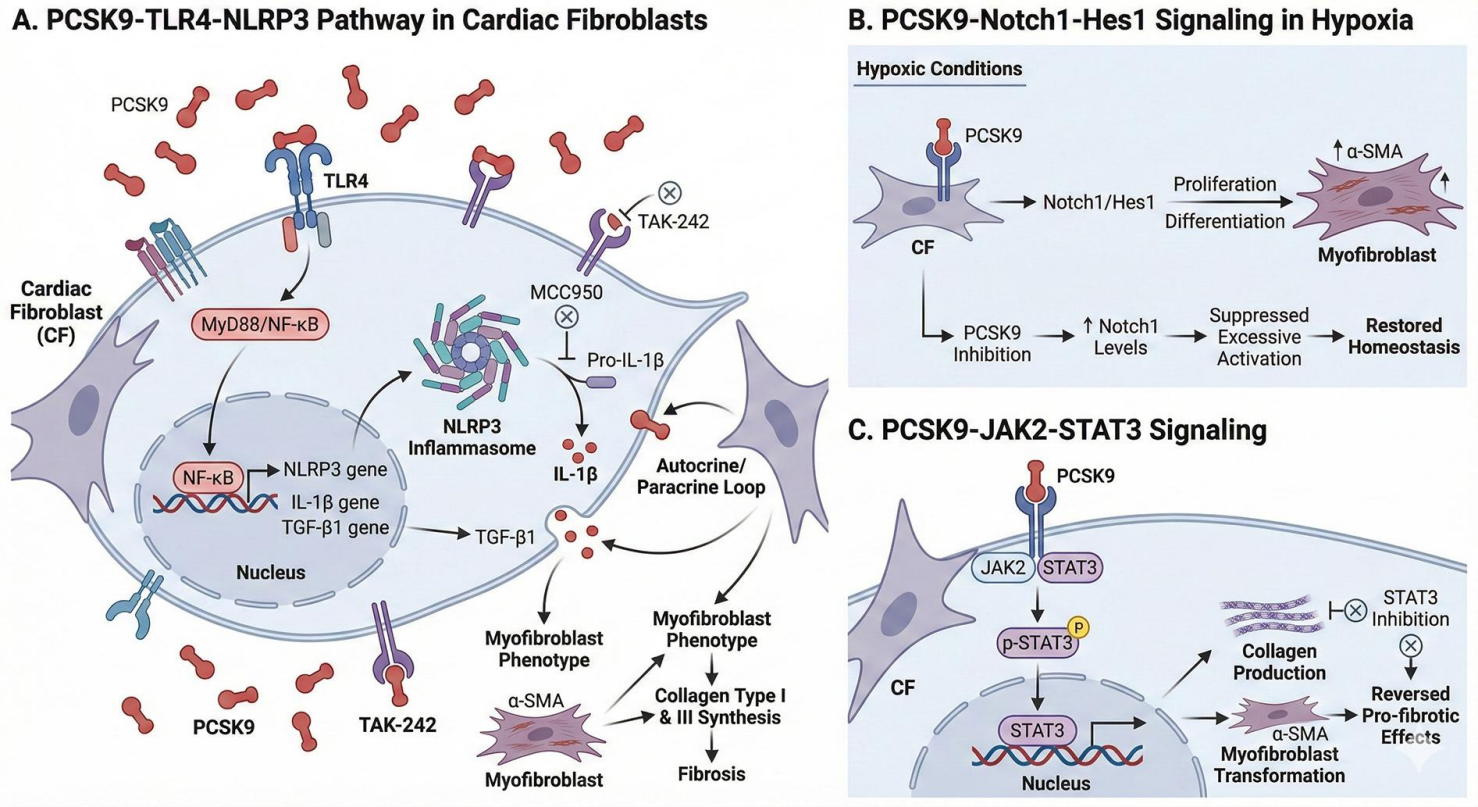
Modulation of cardiac fibroblasts (CFs) by PCSK9. **(A)** PCSK9-TLR4-NLRP3 Pathway: PCSK9 binds to TLR4 on cardiac fibroblasts, activating the MyD88/NF-κB pathway and the NLRP3 inflammasome. This results in the processing and release of mature IL-1β, which acts in an autocrine/paracrine loop to sustain the myofibroblast phenotype (α-SMA expression) and drive collagen synthesis. **(B)** PCSK9-Notch1-Hes1 Signaling in Hypoxia: Under hypoxic conditions, PCSK9 promotes CF proliferation and differentiation into myofibroblasts via the Notch1/Hes1 signaling axis. Inhibition of PCSK9 restores Notch1 levels, suppressing excessive activation and helping to restore homeostasis. **(C)** PCSK9-JAK2-STAT3 Signaling: PCSK9 engages the JAK2/STAT3 pathway, leading to STAT3 phosphorylation and nuclear translocation. This transcriptional activation promotes collagen production and the transformation of fibroblasts into pro-fibrotic myofibroblasts. Created with Biorender.com.

CFs are the primary source of the extracellular matrix. In response to PCSK9, they undergo a phenotypic conversion to myofibroblasts, driving pathological fibrosis that stiffens the ventricle and impairs electrical conduction (67) (Figure 5).

### 6.1 Direct receptor interaction: TLR4 and NLRP3

Crucially, the analysis reveals that CFs express TLR4 and that PCSK9 interacts directly with this receptor to drive fibrosis, independent of lipid metabolism (68).

PCSK9 binding to TLR4 on fibroblasts activates the MyD88/NF-κB pathway. This results in the transcriptional upregulation of NLRP3, IL-1β, and TGF-β1.

The activation of the NLRP3 inflammasome within fibroblasts is a key step. The release of IL-1β acts in an autocrine/paracrine loop to sustain the activated myofibroblast phenotype (characterized by α-SMA expression) and drive collagen type I and III synthesis. Inhibition of NLRP3 (MCC950) or TLR4 (TAK-242) abrogates PCSK9-induced fibrosis, confirming this pathway's centrality.

### 6.2 Differentiation and proliferation

PCSK9 stimulates the proliferation of cardiac fibroblasts and their differentiation into myofibroblasts.

Under hypoxic conditions, PCSK9 promotes CF activation via the Notch1/Hes1 signaling pathway (69). Notch1 signaling is essential for the transition to the myofibroblast state; PCSK9 inhibition increases Notch1 levels, which in this specific ischemic context helps suppress excessive activation (likely by restoring homeostatic Notch signaling that prevents the fibrotic switch).

PCSK9 also engages the JAK2/STAT3 pathway in fibroblasts to promote collagen production and transformation. Inhibition of STAT3 reverses the pro-fibrotic effects of PCSK9 (70).

## 7 The aortic valve: mechanisms of CAVD

**Figure 6:**
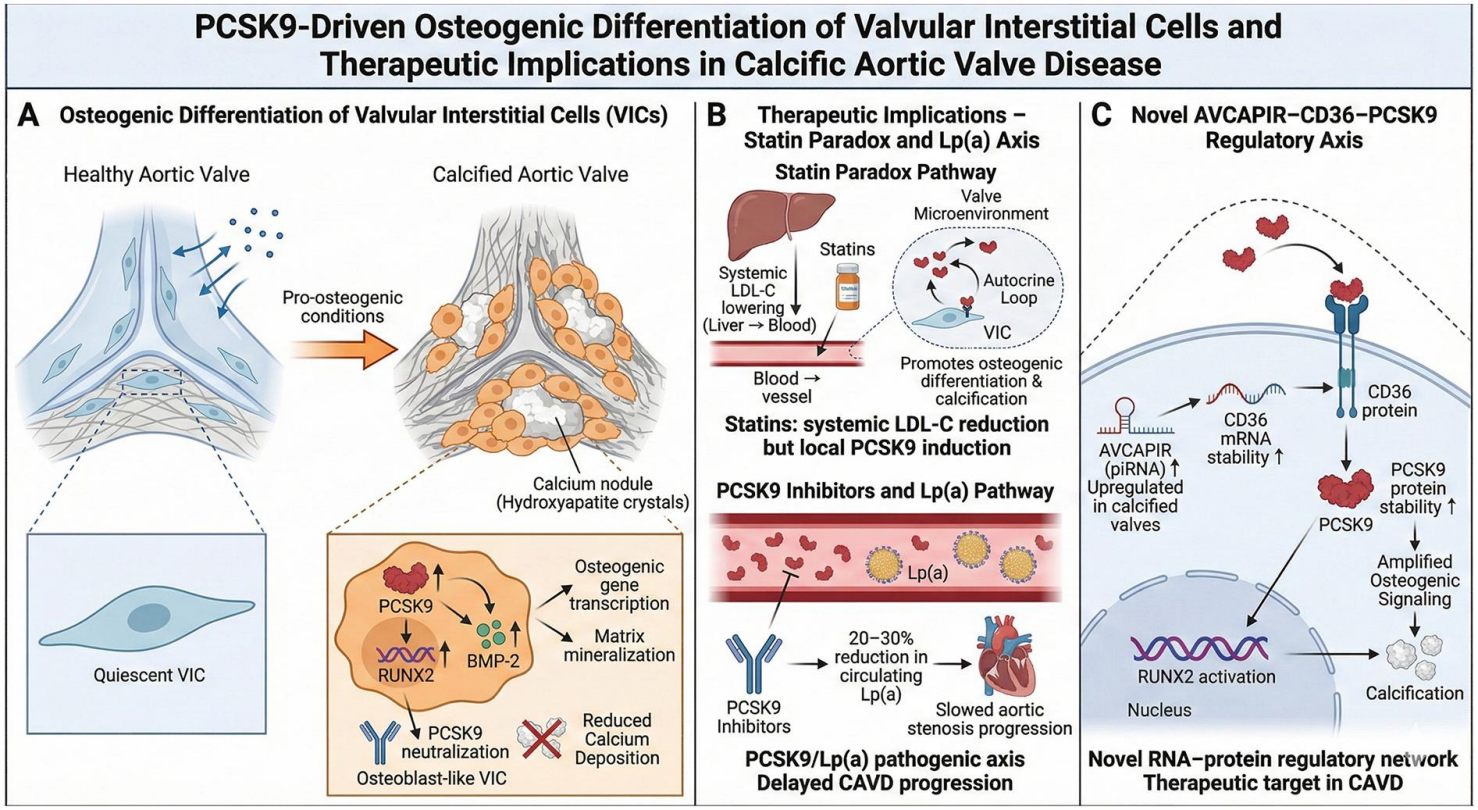
PCSK9-driven osteogenic differentiation of valvular interstitial cells and therapeutic implications in calcific aortic valve disease. **(A)** Osteogenic differentiation of valvular interstitial cells (VICs): Quiescent VICs transition into osteoblast-like cells under pro-osteogenic conditions associated with PCSK9 upregulation. This leads to increased nuclear expression of RUNX2 and BMP-2, driving osteogenic gene transcription, matrix mineralization, and the formation of calcium nodules. **(B)** Therapeutic Implications—Statin Paradox and Lp(a) Axis: Illustrates the “Statin Paradox”, where systemic LDL-C lowering by statins may be counteracted by local statin-induced PCSK9 secretion in the valve, promoting calcification. Conversely, PCSK9 inhibitors reduce both PCSK9 and Lp(a) levels (by 20%–30%), delaying the progression of aortic stenosis. **(C)** Novel AVCAPIR–CD36–PCSK9 Regulatory Axis: A proposed molecular mechanism where the piRNA *AVCAPIR* stabilizes CD36 mRNA. Increased CD36 protein subsequently stabilizes intracellular PCSK9, which amplifies osteogenic signaling and RUNX2 activation, accelerating valvular calcification. Created with Biorender.com.

CAVD, the primary pathology underlying aortic stenosis, is characterized by a complex interplay of lipid infiltration, chronic inflammation, fibrosis, and osteogenic calcification (71, 72). Beyond its established role in atherosclerosis, PCSK9 has emerged as a critical pathogenic driver in CAVD, primarily through its direct actions on VICs to promote osteogenic differentiation and calcification (73) (Figure 6).

### 7.1 Osteogenic differentiation of VICs

The central cellular event in CAVD is the phenotypic transformation of quiescent VICs into osteoblast-like cells.

PCSK9 expression is significantly upregulated in calcified human aortic valves compared to healthy tissue. Under pro-osteogenic conditions *in vitro*, PCSK9 levels rise in human VICs, and PCSK9-ITs markedly attenuates calcium deposition, indicating a direct causal role (73).

PCSK9 promotes the expression of key osteogenic master regulators, including the transcription factor RUNX2 and BMP-2, thereby driving the transcriptional program that facilitates VIC transdifferentiation into a calcifying phenotype.

### 7.2 Therapeutic implications: the statin paradox and the lipoprotein(a) [Lp(a)] connection

The lack of effective pharmacological therapies for CAVD underscores the unique pathophysiology of the valve microenvironment.

Clinical trials have demonstrated that statins, despite robust systemic LDL-C lowering, fail to halt CAVD progression (74, 75). Intriguingly, evidence suggests statins may paradoxically upregulate PCSK9 secretion from VICs in a dose-dependent manner (76). This locally induced PCSK9 then acts in an autocrine fashion to promote osteogenic differentiation, potentially counteracting any beneficial systemic effects within the valve. This paradox highlights PCSK9 inhibitors as a potentially more targeted therapeutic strategy for CAVD.

Elevated PCSK9 and Lp(a) are major independent genetic risk factors for aortic stenosis (77). PCSK9 inhibitors consistently lower Lp(a) levels by 20%–30% (78). Exploratory analyses from large outcome trials (e.g., FOURIER) suggest that PCSK9 inhibition may delay the progression of aortic stenosis, supporting the existence of a contributory PCSK9/Lp(a) pathogenic axis in CAVD (79, 80).

### 7.3 A novel regulator*y* axis: AVCAPIR–CD36–PCSK9

Recent research has identified a specific non-coding RNA pathway driving valvular calcification.A PIWI-interacting RNA(piRNA, AVCAPIR) is upregulated in calcified valves (81). AVCAPIR enhances the stability of CD36 mRNA, leading to increased CD36 protein levels. The increased CD36 subsequently stabilizes PCSK9 protein. This AVCAPIR–CD36–PCSK9 axis potently accelerates the osteogenic transformation of VICs and the progression of CAVD, identifying a novel RNA-protein network ripe for therapeutic targeting.

## 8 Clinical translation and future directions

### 8.1 Evidence from landmark trials and imaging studies

Large-scale cardiovascular outcome trials (CVOTs) have unequivocally established the efficacy of PCSK9-ITs. In the FOURIER and ODYSSEY OUTCOMES trials, evolocumab and alirocumab reduced major adverse cardiovascular events (MACE) by approximately 15% in high-risk statin-treated patients, consistent with the extent of LDL-C lowering (9, 10). Notably, sub-analyses revealed that reductions in Lp(a) independently predicted clinical benefit, and absolute risk reduction was greatest among patients with elevated inflammatory burden, underscoring the contribution of pleiotropic effects (78). Inclisiran, a small interfering RNA (siRNA) therapeutic, has demonstrated durable LDL-C reduction of ∼51% with a favorable safety profile in the ORION program, with definitive outcome data awaited from the ORION-4 trial (7, 8).

Intravascular imaging studies (GLAGOV, PACMAN-AMI, HUYGENS) have provided direct mechanistic insights, showing that PCSK9-ITs promote atheroma regression, thicken fibrous caps, reduce lipid core size, and foster a shift toward more stable, calcified plaque phenotypes (17, 82–85). Despite no significant effect on systemic high-sensitivity C-reactive protein (hs-CRP), these findings confirm potent local anti-inflammatory actions within the plaque, translating into enhanced clinical benefit for patients with high baseline inflammation (54, 86, 87). Collectively, these data position PCSK9-ITs as agents that confer multifaceted cardiovascular protection through profound LDL-C reduction and direct plaque stabilization.

### 8.2 Unresolved controversies: the efficacy gap and inhibitor equivalence

A central, unresolved question is whether pharmacological PCSK9-ITs fully recapitulates the profound cardioprotection observed in individuals with lifelong genetic PCSK9 deficiency, who exhibit up to an 88% reduction in coronary heart disease risk (4). The more modest relative risk reduction (∼15%) seen in CVOTs suggests a potential “efficacy gap” (8–10).

Two non-mutually exclusive hypotheses may explain this discrepancy. The first posits the critical role of cumulative LDL-C exposure in atherosclerosis pathogenesis. Mendelian randomization studies indicate that the magnitude of CVD risk reduction depends on both the extent and the duration of LDL-C lowering (88–91). Thus, the lifelong low LDL-C in genetic carriers may confer superior protection compared to the relatively shorter-term pharmacological reduction in trials.

The second hypothesis concerns structural and functional differences in inhibition strategies. Monoclonal antibodies (e.g., evolocumab, alirocumab) block the PCSK9 catalytic domain, preventing LDLR degradation but leaving the CHRD intact and capable of propagating inflammation through receptors like CAP1 (20, 42, 92, 93). In contrast, genetic knockout ablates the entire protein, silencing both canonical and non-canonical pathways. This distinction is supported by preclinical data showing that genetic knockout, but not antibody treatment, protects against post-myocardial infarction mortality. Consequently, therapeutic modalities that suppress protein synthesis more completely (e.g., siRNA like inclisiran) or eliminate expression permanently (e.g., gene editing) may more closely approximate the benefits of genetic deficiency, a consideration particularly relevant for patients with high residual inflammatory risk.

### 8.3 Next-generation therapeutic strategies

The therapeutic landscape for PCSK9-ITs is rapidly expanding, focusing on improved convenience, accessibility, and completeness of target engagement.

Oral Small-Molecule Inhibitors: The development of orally bioavailable inhibitors represents a major advance. MK-0616 demonstrated dose-dependent LDL-C reductions up to 60.9% in a Phase 2b trial, with positive Phase 3 results recently announced (94). Similarly, AZD0780, which uniquely targets the CHRD without disrupting the PCSK9–LDLR interaction, achieved a 50.7% LDL-C reduction in the Phase 2b PURSUIT trial (95). These agents promise efficacy comparable to injectables in a convenient oral form, with ongoing outcome trials set to define their clinical role.

Therapeutic Vaccines: Active immunization strategies aim to induce durable, endogenous anti-PCSK9 antibodies. Vaccine candidates like VXX-401 and AT04A have shown promise in preclinical and early-phase clinical studies, eliciting immune responses and sustaining modest LDL-C reductions (e.g., 11%–13% with AT04A) (96, 97). Key challenges include inter-individual variability in immune response and the need for booster immunizations.

Gene Editing: *in vivo* gene editing (e.g., VERVE-101/102) seeks to permanently inactivate the PCSK9 gene via CRISPR-Cas9 base editing. Preliminary data from the Heart-1 trial are striking: a single infusion of VERVE-101 in patients with heterozygous familial hypercholesterolemia produced durable, dose-dependent reductions in PCSK9 and LDL-C (up to 55% at 6 months) (98, 99). While heralding a potential “one-and-done” cure, this irreversible approach necessitates meticulous long-term safety evaluation regarding off-target effects and potential consequences of lifelong PCSK9 ablatio.

Notably, emerging evidence complicates the assumption that maximal PCSK9 suppression is uniformly beneficial. Lifelong, complete PCSK9 deficiency in murine models induces an HFpEF-like phenotype characterized by diastolic dysfunction, myocardial lipid accumulation, and mitochondrial impairment, independent of circulating PCSK9 levels (82). This contrasts with pathological states in which elevated PCSK9 exacerbates ischemic injury and adverse remodeling, contributing to HFrEF (100). Together, these findings suggest a bimodal, context-dependent role for PCSK9 in cardiac physiology, potentially mediated by altered myocardial lipid handling through CD36 upregulation. Consequently, maintaining physiologically low—but not absent—PCSK9 activity may be optimal for long-term cardiac health. This paradigm has important implications for next-generation therapies, particularly permanent gene-editing strategies aimed at complete PCSK9 ablation.

### 8.4 Future perspectives and recommendations

Despite the successful clinical translation of PCSK9-ITs for LDL-C lowering, accumulating evidence indicates that PCSK9 functions as a pleiotropic regulator of cardiovascular pathophysiology through multiple LDLR-independent mechanisms. These observations open several important avenues for future research and therapeutic development.

First, dissecting pathway-specific inhibition remains a priority. Current monoclonal antibodies primarily block the interaction between PCSK9 and LDLR, while leaving the CHRD intact. As the CHRD mediates key inflammatory and immune effects through receptors such as CAP1, CD36, and MHC-I, next-generation inhibitors capable of neutralizing these non-canonical interactions may better address residual inflammatory and thrombotic risk.

Second, patient stratification based on non-lipid risk profiles warrants further investigation. Clinical and imaging data suggest that individuals with elevated inflammatory burden, enhanced platelet reactivity, high Lp(a), or early valvular calcification may derive disproportionate benefit from PCSK9-ITs beyond LDL-C reduction. Biomarker-guided strategies could therefore refine patient selection and optimize therapeutic yield.

Third, long-term safety of profound PCSK9 suppression requires careful evaluation. Preclinical data indicating metabolic and myocardial consequences of complete PCSK9 deficiency raises important considerations for permanent gene-editing approaches. Future studies should distinguish between partial, reversible inhibition and lifelong ablation, particularly in relation to myocardial metabolism and heart failure phenotypes.

Fourth, expanding indications beyond atherosclerosis represents a promising direction. The involvement of PCSK9 in thrombosis, myocardial injury and CAVD suggests therapeutic potential in acute coronary syndromes, heart failure remodeling, and valvular heart disease—areas where effective disease-modifying pharmacotherapies remain limited.

Finally, integration of novel therapeutic platforms, including oral small-molecule inhibitors, vaccines, and gene-editing technologies, offers unprecedented opportunities to tailor the depth, duration, and tissue specificity of PCSK9 inhibition. Comparative studies assessing how these modalities differentially modulate LDLR-dependent and LDLR-independent pathways will be essential for defining their optimal clinical roles.

## 9 Conclusion

This article provides a systematic review of the non-classical biological functions of PCSK9 within the cardiovascular system. It highlights that beyond regulating cholesterol metabolism via degradation of the LDLR, PCSK9 also directly contributes to key pathological processes through multiple LDLR-independent pathways. These processes include vascular inflammation, atherosclerosis progression, thrombosis, myocardial injury and remodeling and aortic valve calcification. Current basic and clinical evidence indicates that the cardiovascular benefits of PCSK9-ITs cannot be attributed solely to LDL-cholesterol reduction; its anti-inflammatory, antithrombotic, and tissue-protective effects represent significant independent mechanisms. A deeper understanding of the pleiotropic actions of PCSK9 will help optimize patient stratification, expand therapeutic indications, and provide a rationale for developing next-generation therapies targeting non-LDLR pathways.
